# EuroScore and IL-6 predict the course in ICU after cardiac surgery

**DOI:** 10.1186/s40001-021-00501-1

**Published:** 2021-03-26

**Authors:** Andreas Bauer, Insa Korten, Gerd Juchem, Isabel Kiesewetter, Erich Kilger, Jens Heyn

**Affiliations:** 1 Department of Anaesthesiology, University of Munich (LMU), Wolkerweg 16, 81375 Munich, Germany; 2 Department of Anesthesiology, Klinikum Rosenheim, Pettenkoferstraße 10, 83022 Rosenheim, Germany; 3 Division of Respiraotry Medicine, Department of Pediatrics, Inselspital and University of Bern, 3010 Bern, Switzerland; 4 Department of Cardiac Surgery, University of Munich (LMU), Wolkerweg 16, 81375 Munich, Germany; 5 Department of Anaesthesiology, University of Munich (LMU), Marchioninistrasse 15, 81377 Munich, Germany

**Keywords:** Cardiac surgery, Intensive care medicine, IL-6, EuroScore, Outcome

## Abstract

**Background:**

Despite modern advances in intensive care medicine and surgical techniques, mortality rates in cardiac surgical patients are still about 3%. Considerable efforts were made to predict morbidity and mortality after cardiac surgery. In this study, we analysed the predictive properties of EuroScore and IL-6 for mortality in ICU, prolonged postoperative mechanical ventilation, and prolonged stay in ICU.

**Methods:**

We enrolled 2972 patients undergoing cardiac surgery. The patients either underwent aortic valve surgery (AV), mitral valve surgery (MV), coronary artery bypass grafting (CABG), and combined operations of aortic valve and coronary artery bypass grafting (AV + CABG) or of mitral and tricuspid valve (MV + TV). Different laboratory and clinical parameters were analysed.

**Results:**

EuroScore as well as IL-6 were associated with increased mortality after cardiac surgery. Furthermore, a higher EuroScore and elevated levels of IL-6 were predictors for prolonged mechanical ventilation and a longer stay in ICU. Especially, highly significant elevated IL-6 levels and an increased EuroScore showed a strong association. Statistics suggested superiority when both parameters were combined in a single model.

**Conclusion:**

Our results suggest that EuroScore and IL-6 are helpful in predicting the course in ICU after cardiac surgery, and therefore, the use of intensive care resources. Especially, the combination of highly elevated levels of IL-6 and EuroScore may prove to be excellent predictors for an unfortunate postoperative course in ICU.

**Supplementary Information:**

The online version contains supplementary material available at 10.1186/s40001-021-00501-1.

## Introduction

Over the last decades, an astonishing progress was registered in cardiac surgery [1]. Operations of most complex cardiovascular pathologies and abnormalities have become somehow routine nowadays [2]. Despite modern advances in intensive care medicine and surgical techniques, mortality rates (within 30 days after surgery) in all cardiac surgical patients are still about 3% [3, 4].

Considerable efforts were made to predict postoperative morbidity and mortality after cardiac surgery in the past [5–7]. Several studies attempted to find single predictors or developed algorithms and scoring systems to identify patient characteristics with an increased risk of an adverse outcome after surgery [8, 9]. One of the most established predictive models is the European System for Cardiac Operative Risk Evaluation (EuroScore) [9]. The EuroScore is a simple-to-use, objective, and well validated scoring system to predict early mortality after cardiac surgery [9]. Patient characteristics, as well as cardiac and operation related risk variables are collected and weighted differently within this model [9]. It considers previous cardiac surgery, emergency surgery other than isolated coronary artery bypass grafting (CABG), and surgery on thoracic aorta [9]. The use of the EuroScore to predict mortality (30 days from operation or later than 30 days if the patient is still in the hospital) is well documented in the literature [10]. Its association with postoperative parameters is less well investigated. An association of the EuroScore and the length of stay in ICU after cardiac surgery was shown in a recent research conducted at a single centre in the UK [11]. The role of EuroScore to predict other postoperative parameters like length of mechanical ventilation seems to be unknown.

Recent research showed that systemic inflammation after cardiac surgery is associated with an increased mortality [12]. While the EuroScore considers several important clinical parameters, the inflammatory response after cardiac surgery is not included. The inflammatory response in the setting of cardiac surgery can most likely be explained by surgical trauma, cardiopulmonary bypass, and reperfusion injury [13, 14]. Interleukin-6 (IL-6) is induced by tumour necrosis factor (TNF)-α and reflects the localized TNF-α activity suggesting a major role of TNF-α and IL-6 in the inflammatory response after cardiac surgery [13, 14]. However, the association of IL-6 with short-term outcome after cardiac surgery has not been investigated yet.

Therefore, the aim of our study was to investigate the association and possible predictive properties of (i) the EuroScore and (ii) IL-6 serum levels for mortality in ICU, prolonged postoperative mechanical ventilation, and prolonged stay in ICU after cardiac surgery.

## Material and methods

The study followed the principles of the Declaration of Helsinki and was approved by the Institutional Review Board of the Ludwig Maximilians University Munich (approval number: 748-15).

### Patients

We retrospectively analysed the records of 2972 patients who underwent either CABG, aortic valve surgery (AV), combined operation of aortic valve and coronary artery bypass grafting (AV + CABG), mitral valve surgery (MV), and combined operation of mitral and tricuspid valve (MV + TV) at the University of Munich (LMU; Herzklinik der Universität München am Augustinum) over a period of 5 years. None of the patients had signs of infection in either clinical examination or laboratory testing.

### Recorded parameters

The documented parameters analysed in this study were: age, sex, ejection fraction (EF) and serum creatinine level before operation, duration of cardiopulmonary bypass (CPB), length of stay in the ICU, length of mechanical ventilation, serum levels of interleukin 6 (IL-6) 6 h after operation, additive EuroScore (calculated during the pre-operative assessment), and death after cardiac surgery.

### ICU procedures

All operations were performed under extracorporeal circulation. Clinical management (mechanical ventilation, extubation, fluid management, catecholamine support, and discharge from the ICU) was performed according to a standardized protocol. All patients were mechanically ventilated at admission to the ICU. Tracheal extubation was performed after a period of spontaneous breathing according to the following criteria: absence of residual muscle paralysis, adequate ventilatory parameters, patient awake, and cooperative. Fluid therapy was guided by transoesophageal echocardiography and haemodynamic monitoring. Normovolemia was achieved by infusions of crystalloids and colloids.

Discharge from the ICU required the following criteria: hemodynamic stability without pharmacological support, adequate gas exchange, no signs of bleeding or systemic infections, and adequate urine output.

### Statistical analyses

In a first step, we analysed different possible predictors for mortality in ICU after surgery. Investigated variables were the EuroScore and IL-6 serum levels. Furthermore, we included age (linear in years), sex, pre-operative serum creatinine levels, duration of cardiopulmonary bypass, EF, and kind of cardiac surgery. For this, we fitted univariable logistic regression models with death as outcome variable. In a second step, we investigated different possible predictors for (a) duration of ventilation after surgery and (b) duration of stay in ICU after surgery. We included the same exposure variables as listed above. We fitted univariable and multivariable logistic regression models. In multivariable models we further tested for interactions between EuroScore and IL-6 serum levels and the other included variables using likelihood ratio tests. As the EuroScore could not be obtained in a small number of patients, we performed sensitivity analyses, repeating analyses for the other variables in patients (a) where the Euroscore was obtained and (b) without EuroScore. Variables with a *p* value ≤ 0.05 (likelihood ratio test) were considered as statistically significant. Multivariable models were compared with the likelihood ratio test. All statistical analyses were performed using Stata™ (Stata Statistical Software: Release 13. College Station, TX: StataCorp LP).

## Results

### Study population

We analysed data from 2972 patients (2105 male, approximately 71%) undergoing cardiac surgery. Of those, 1568 patients underwent CABG, 729 AV, 359 AV + CABG, 262 MV, and 54 MV + TV, respectively. After surgery, patients were transferred to ICU. The median interquartile range (IQR) stay in ICU for all patients was 2 (1–4) days and the median (IQR) of mechanical ventilation was 12 (8–18) hours after surgery. Amongst 2972 patients, 40 (1.3%) died in ICU after surgery. The 30-day mortality was 1.6% (49 patients), the 90-day mortality was 1.9% (56 patients). Spearmen correlation did not show a significant correlation between age and IL-6 levels 6 h after surgery (*r* = 0.005; *p* = 0.776). For details, see Table 1.

**Table 1:** Characteristics of the study population

	Patients (*n* = 2972)
Anthropometrics	
Sex, males	2105 (70.8)
Age [years]^a^	72 (64–78)
Death after surgery [*n*]	40 (1.3)
Intensive care treatment	
Duration of ventilation [hours]^a^	12 (8–18)
Length of stay in ICU [days]^a^	2 (1–4)
EuroScore	
Low risk (0–2)	800 (27.4)
Medium risk (3–5)	1176 (39.6)
High risk (> 6)	871 (29.3)
Laboratory assessment^a^	
IL-6 [pg/ml]	206 (116–364)
Creatinine [mg/dl]	1 (0.9–1.2)
EF [%]^a^	65 (55–65)
CPB [min]^a^	92 (75–117)
Surgery	
CABG	1568 (52.8)
AV	729 (24.5)
CABG + AV	359 (12.1)
MV	262 (8.8)
MV + TV	54 (1.8)

Results are displayed in numbers (%) if not stated otherwise. ^a^Results are displayed as median (IQR)

*ICU* intensive care unit, *EF* ejection fraction, *CPB* cardiopulmonary bypass, *CABG* coronary artery bypass grafting, *AV* aortic valve surgery, *CABG* + *AV* combined operation of aortic valve and coronary artery bypass grafting, *MV* mitral valve surgery, *MV* + *TV* combined operation of mitral and tricuspid valve

### Possible predictors for mortality in ICU after cardiac surgery

We investigated the association of the EuroScore and IL-6 serum levels with other possible predictors for mortality in ICU after cardiac surgery. In the univariable models, a higher EuroScore and higher IL-6 levels were associated with mortality in ICU after surgery. For IL-6, only the highest quintile (> 421 pg/ml) reached the significance level. In addition, older age, elevated pre-operative serum creatinine, longer CPB time and lower EF, as well as kind of surgery (with a lower risk for CABG and AV and the highest risk after MV and TV replacement) were associated with mortality in ICU after surgery. The results are summarized in more detail in Table 2.

**Table 2:** Investigation of possible predictors for mortality after cardiac surgery

Variable	Category	OR	95% CI	*p *value^h^
Age^a^	Linear	1.08	[1.03, 1.12]	0.001
Sex^b^	Male	1	[1.00, 1.00]	0.03
	Female	1.46	[0.77, 2.79]	
EF [%]^c^	25–45	1	[1.00, 1.00]	0.0003
	46–64	0.19	[0.07, 0.57]	
	65	0.3	[0.16, 0.58]	
EuroScore^d^	0–2	1	[1.00, 1.00]	< 0.001
	3–5	2.05	[0.41, 10.17]	
	> 6	13.25	[3.15, 55.81]	
Creatinine [mg/dl]^e^	< 1.2	1	[1.00, 1.00]	< 0.001
	1.2–2.1	5.6	[2.67, 11.76]	
	> 2.1	13.09	[4.66, 36.81]	
IL-6 [pg/ml]^f^	Q1 (< 102)	1	[1.00, 1.00]	< 0.001
	Q2 (102–165)	1.51	[0.25, 9.09]	
	Q3 (166–256)	2.01	[0.37, 11.02]	
	Q4 (257–421)	2.03	[0.37, 11.11]	
	Q5 (> 421)	12.6	[2.96, 53.55]	
CPB [min]^g^		2.3	1.66, 3.15	< 0.001

Logistic regression model investigating possible predictors for death (yes/no) after cardiac surgery. Only results from the univariable models are displayed

*EF* ejection fraction, *CPB* cardiopulmonary bypass, *OR* odds ratio, *CI* confidence interval

^a^Age is analysed as linear variable

^b^The reference category are male

^c^EF displayed in quintiles (resulting in 3 groups), reference category are values 45

^d^EuroScore is analysed in three groups according to guidelines: mild/moderate/severe risk. The reference category is the lowest score

^e^Creatinine is displayed in three groups. The reference category are lowest values

^f^IL-6 is displayed in quintiles. The reference category is the first quintile

^g^CPB. Due to the low (missing) number of deaths in the lower quintiles, overall OR is displayed

^h^*p* value: from likelihood ratio test

### Possible predictors for prolonged mechanical ventilation and length of stay in ICU after cardiac surgery

In an additional analysis, we investigated the known risk factors from the primary analysis (above) and chose as outcome parameters (a) duration of ventilation (in hours, dividing the patients into two sub-groups, less or more than 12 h) and (b) duration of treatment in ICU (in days, again dividing the patients into 2 sub-groups, less or more than 2 days). In univariable analyses, we could confirm that both outcome parameters, the EuroScore and IL-6 serum levels as well as the other predictors for mortality in ICU after surgery (Table 2) were associated with the duration of ventilation and the length of stay in ICU (Tables 3, 4, respectively). IL-6 measures did not reach significance level in the univariable model for duration of stay in ICU, however, confidence intervals showed a difference for the sub-group with highest IL-6 levels. To identify possible confounding factors, we then included all selected parameters in a multivariable model. Results of all selected parameters except sex were consistent with the univariable analyses, thus suggesting that all these parameters are associated with duration of ventilation and length of stay in ICU. The type of surgery was not associated with the time of ventilation in this setting. Taking a closer look at IL-6 measures, association strengthened further in the multivariable analyses, with strong association observed not only in the highest quintile of IL-6 levels, but already in the lower quantiles (Tables 3, 4, respectively). We observed that the duration of mechanical ventilation and length of stay increased in patients with a combination of higher EuroScore and higher IL-6 measures, than elevated EuroScore and IL-6 measures alone (regression model including interaction terms, likelihood ratio test: *p* = 0.01 and *p* = 0.003, respectively, details are provided in Additional file 1: Table S1 and Additional file 2: Table S2). As the EuroScore could not be obtained in 125 patients, we performed sensitivity analyses for the other variables in patients (a) with EuroScore and (b) without EuroScore. After adjustment for confounders, results remained robust in all analyses, indicating that the EuroScore and IL-6 measures alone and combined, were excellent predictors for all investigated outcomes.

**Table 3:** Investigation of possible predictors for duration of ventilation after cardiac surgery

Variable	Category	Univariable model			Multivariable model		
		OR	95% CI	*p* value^j^	OR	95% CI	*p* value^j^
Age [years]^a^	Linear	1.03	[1.02, 1.04]	< 0.001	1.01	[1.00, 1.02]	0.004
Sex^b^	Male	1	[1.00, 1.00]	0.066	1	[1.00, 1.00]	0.15
	Female	1.16	[0.99, 1.36]		1.15	[0.95, 1.38]	
EF [%]^c^	≤ 45	1	[1.00, 1.00]	0.015	1	[1.00, 1.00]	0.004
	46–64	0.76	[0.61, 0.95]		1.02	[0.80, 1.29]	
	≥ 65	0.59	[0.49, 0.71]		0.77	[0.62, 0.95]	
EuroScore^d^	0–2	1	[1.00, 1.00]	< 0.001	1	[1.00, 1.00]	< 0.001
	3–5	1.56	[1.29, 1.88]		1.29	[1.03, 1.62]	
	> 6	3.05	[2.50, 3.74]		2.07	[1.58, 2.72]	
Creatinine [mg/dl]^e^	< 1.2	1	[1.00, 1.00]	< 0.001	1	[1.00, 1.00]	< 0.001
	1.2–2.1	1.73	[1.48, 2.04]		1.54	[1.29, 1.83]	
	> 2.1	2.99	[1.93, 4.63]		1.9	[1.18, 3.05]	
IL-6 [pg/ml]^f^	Q1 (< 102)	1	[1.00, 1.00]	0.009	1	[1.00, 1.00]	0.003
	Q2 (102–165)	1.01	[0.80, 1.427]		1.16	[0.91, 1.49]	
	Q3 (166–256)	1.16	[0.92, 1.46]		1.35	[1.05, 1.73]	
	Q4 (257–421)	1.11	[0.88, 1.40]		1.39	[1.07, 1.80]	
	Q5 (> 421)	1.45	[1.16, 1.83]		1.79	[1.38, 2.32]	
CPB [min]^g^	Q1 (< 72)	1	[1.00, 1.00]	< 0.001	1	[1.00, 1.00]	< 0.001
	Q2 (72–85)	1	[0.80, 1.27]		0.97	[0.76, 1.24]	
	Q3 (86–100)	1.04	[0.82, 1.31]		0.97	[0.75, 1.24]	
	Q4 (101–124)	1.16	[0.92, 1.46]		1.09	[0.85, 1.40]	
	Q5 (> 124)	1.83	[1.45, 2.30]		1.66	[1.27, 2.16]	
Operation^h^	CABG	1	[1.00, 1.00]	< 0.001	1	[1.00, 1.00]	0.53
	AV	0.99	[0.83, 1.018]		1.01	[0.83, 1.24]	
	CABG + AV	1.43	[1.13, 1.80]		1.08	[0.82, 1.43]	
	MV	1.13	[0.87, 1.47]		1.08	[0.80, 1.45]	
	MV + TV	3.26	[1.78, 5.97]		1.78	[0.91, 3.51]	

Logistic regression model investigating possible predictors for duration of ventilation after cardiac surgery. Baseline are patients with a ventilation time ≤ 12 h. The univariable model are shown in the left columns, the multivariable model adjusted to all variables listed above is presented in the right columns

^a^Age is analysed as linear variable

^b^The reference category are males

^c^Ejection fraction (EF) displayed in quintiles (resulting in 3 groups), reference category are values ≤ 45

^d^EuroScore analysed in three groups according to guidelines: mild/moderate/severe risk. The reference category is the lowest score

^e^Creatinine displayed in three groups. The reference category are lowest values

^f^IL-6 is displayed in Q (quintiles). The reference category is the first quintile

^g^CPB is displayed in Q (quintiles). The reference category is the first quintile

^h^Reference category is CABG

^j^*p* value: from likelihood ratio test

*OR* odds ratio, *CI* confidence interval, *EF* ejection fraction, *CPB* cardiopulmonary bypass, *CABG* coronary artery bypass grafting, *AV* aortic valve surgery, *CABG* + *AV* combined operation of aortic valve and coronary artery bypass grafting, *MV* mitral valve surgery, *MV* + *TV* combined operation of mitral and tricuspid valve

**Table 4:** Investigation of possible predictors for duration of stay in ICU after surgery

Variable	Category	Univariable model			Multivariable model		
		OR	95% CI	*p* value^j^	OR	95% CI	*p* value^j^
Age [years]^a^	Linear	1.02	[1.01, 1.03]	< 0.001	1.01	[1.00, 1.02]	0.02
Sex^b^	Male	1	[1.00, 1.00]	0.6	1	[1.00, 1.00]	0.8
	Female	1.04	[0.89, 1.22]		1.03	[0.85, 1.24]	
EF [%]^c^	≤ 45	1	[1.00, 1.00]	< 0.001	1	[1.00, 1.00]	0.001
	46–64	0.56	[0.45, 0.70]		0.71	[0.56, 0.90]	
	≥ 65	0.53	[0.44, 0.63]		0.68	[0.56, 0.84]	
EuroScore^d^	0–2	1	[1.00, 1.00]	< 0.001	1	[1.00, 1.00]	< 0.001
	3–5	1.37	[1.14, 1.66]		1.29	[1.03, 1.61]	
	> 6	2.61	[2.14, 3.18]		1.97	[1.50, 2.59]	
Creatinine [mg/dl]^e^	< 1.2	1	[1.00, 1.00]	< 0.001	1	[1.00, 1.00]	< 0.001
	1.2–2.1	1.76	[1.50, 2.07]		1.56	[1.31, 1.86]	
	> 2.1	2.5	[1.64, 3.80]		1.71	[1.07, 2.72]	
IL-6 [pg/ml]^f^	Q1 (< 102)	1	[1.00, 1.00]	0.2	1	[1.00, 1.00]	0.009
	Q2 (102–165)	1.18	[0.94, 1.48]		1.41	[1.10, 1.81]	
	Q3 (166–256)	1.17	[0.93, 1.47]		1.3	[1.01, 1.67]	
	Q4 (257–421)	1.12	[0.89, 1.41]		1.31	[1.01, 1.70]	
	Q5 (> 421)	1.33	[1.05, 1.67]		1.6	[1.23, 2.07]	
CPB [min]^g^	Q1 (< 72)	1	[1.00, 1.00]	< 0.001	1	[1.00, 1.00]	< 0.001
	Q2 (72–85)	1.18	[0.93, 1.48]		1.15	[0.90, 1.47]	
	Q3 (86–100)	0.99	[0.78, 1.25]		0.9	[0.70, 1.16]	
	Q4 (101–124)	1.2	[0.95, 1.51]		1.04	[0.81, 1.33]	
	Q5 (> 124)	2.29	[1.82, 2.89]		1.92	[1.47, 2.50]	
Operation^h^	CABG	1	[1.00, 1.00]	< 0.001	1	[1.00, 1.00]	0.02
	AV	0.84	[0.70, 1.00]		0.89	[0.73, 1.08]	
	CABG + AV	1.17	[0.93, 1.47]		0.89	[0.67, 1.17]	
	MV	1.29	[0.99, 1.67]		1.28	[0.95, 1.72]	
	MV + TV	3.67	[1.98, 6.81]		2.19	[1.09, 4.36]	

Logistic regression model investigating possible predictors for duration of stay in ICU. Baseline are patients being dismissed from ICU ≤ 2 days (compared to patients with being hospitalized on ICU > 2 days). The univariable model is shown on the left, the multivariable model on the right

^a^Age is analysed as linear variable

^b^The reference category are males

^c^Ejection fraction (EF) displayed in quintiles (resulting in 3 groups), reference category are values ≤ 45

^d^EuroScore analysed in three groups according to guidelines: mild/moderate/severe risk. The reference category is the lowest score

^e^Creatinine displayed in three groups. The reference category are lowest values

^f^IL-6 is displayed in Q (quintiles). The reference category is the first quintile

^g^CPB is displayed in Q (quintiles). The reference category is the first quintile

^h^Reference category is CABG

^j^*p* value: from likelihood ratio test

*OR* odds ratio, *CI* confidence interval, *EF* ejection fraction, *CPB* cardiopulmonary bypass, *CABG* coronary artery bypass grafting, *AV* aortic valve surgery, *CABG* + *AV* combined operation of aortic valve and coronary artery bypass grafting, *MV* mitral valve surgery, *MV* + *TV* combined operation of mitral and tricuspid valve

## Discussion

Prediction of morbidity and mortality after cardiac surgery during the stay in ICU is a very important issue for the patients, for the management of an ICU, and for the relatives of the patients. Furthermore, predictors should also be able to guide clinicians in making recommendations about an operation by helping them to weigh the risks against the benefits. These predictors may also serve as an instrument of quality control. In our study, the EuroScore as well as IL-6 measures were associated with an increased mortality after cardiac surgery. Furthermore, a higher EuroScore (calculated before operation) and significant elevated IL-6 levels (measured 6 h after operation) were predictors for prolonged mechanical ventilation and a longer stay in ICU.

In his work, Meadows et al. [11] showed the predictive value of the EuroScore in terms of length of stay in ICU. Since this evaluation has been performed in a single centre in the UK, these findings cannot be generally applied to patients outside the UK without further investigation. However, observations made by Meadows et al. [11] are consistent with the findings in our trial conducted with a large sample size of 2972 patients residing in Germany. While Meadows et al. [11] analysed the value of the EuroScore in predicting the length of stay in ICU after cardiac surgery, our analyses could also show the potential of the EuroScore in predicting the length of mechanical ventilation. This is a very important finding especially when there are limited resources for mechanical ventilation. During the COVID-19 pandemic, when many hospitals faced a severe shortage of resources for the mechanical ventilation [15], an instrument that helps to manage the usage of respirators is extremely valuable.

Another interesting and surprising finding of our analyses was that the variable, gender (female) is associated with an increased mortality without being associated with prolonged mechanical ventilation. The findings in our study support a well-known phenomenon in cardiac surgery, namely that women have higher morbidity and mortality after cardiac surgery than men. Different explanations for this phenomenon have been suggested. This includes anatomically smaller coronary arteries in the female heart, the older age of women undergoing cardiac surgery, a higher likelihood of unstable angina and higher rates of comorbidities in women [16, 17]. In general, scientific data supporting these explanations are still rare. One possible approach is seen in a higher risk for new neurological events after cardiac surgery as a cause for increased 30-day mortality in women undergoing cardiac surgery [18]. The scientific discussion related to the influence of female gender on prolonged mechanical ventilation after surgery is controversial [19]. Some work favours female gender as a risk factor in these circumstances, other suggest no influence of the female gender on prolonged mechanical ventilation [19]. In our study, female gender was not a predictor for the length of mechanical ventilation. To address this question in more detail, further research has to be performed with this specific scientific question.

Several risk models have been developed to predict mortality after cardiac surgery [20]. One of the best-known risk models for patients undergoing cardiac surgery is the EuroScore [9]. The EuroScore was initially developed to predict the peri-operative mortality in patients undergoing CABG [9]. It is known that different versions of the EuroScore exist, an additive and a logistic version. The additive version is easier and more practical to use than the logistic form [21]. There is still an ongoing controversy concerning the possible advantages of one model over the other [21]. Depending on the analysed patient group and the scientific issue, researchers found no advantage of using the logistic over the additive model [22], others found advantages of the logistic model in high-risk patients [23]. However, the additive EuroScore has been validated as predictor for the postoperative course after cardiac surgery [11, 24–26]. Since it is more convenient to use, the additive EuroScore, we used this version in our study. We could show that the additive EuroScore is also a good predictor for the length of stay in ICU and for the duration of mechanical ventilation.

Off-pump cardiac procedures are associated with decrease in inflammatory response and postoperative morbidity, however, CPB is still inevitable in a large number of surgical procedures [27, 28]. Notwithstanding the tireless development and improvement of CPB, CPB still leads to activation of the coagulation, fibrinolytic, and inflammatory system [29–31]. These pathophysiological changes are most reasonably caused by the exposition of the blood to the artificial surface of the bypass circuit [32]. The consequence is frequently a degranulation of leukocytes and a release of cytotoxic and inflammatory mediators such as interleukins [29–31]. Lymphocytes, fibroblasts, macrophages, and endothelial cells are the main source for secretion of IL-6 in humans [33]. IL-6 itself is an important proinflammatory mediator well characterized in the orchestration of the inflammatory processes [34]. It has been suggested that the myocardium produces IL-6 during phases of compromised myocardial function as a result of ischemia and reperfusion injuries [35]. Furthermore, the surgical trauma might also be reasonable for an increase of IL-6 within the first 4 to 6 h after surgery [35, 36]. These close interlockings of IL-6, surgical trauma and myocardial function makes IL-6 an interesting parameter for predicting the outcome of cardiac surgery in ICU. Indeed, our analyses showed that especially IL-6 serum levels 6 h after operation could be a useful predictor for mortality, prolonged mechanical ventilation, and prolonged stay in ICU after cardiac surgery. IL-6 measures therefore might be a helpful parameter to optimize the management of postoperative processes and planning in ICU. Interestingly, the combination of IL-6 and EuroScore might even improve the prediction of death, prolonged mechanical ventilation, and prolonged stay in ICU after cardiac surgery. Therefore, one should consider including the inflammatory response after operations (e.g., IL-6 6 h after operations) when revising the EuroScore.

## Strength and limitations

Strength of our study is the large dataset of patients and the large number of various patient-related variables that could be included. Furthermore, we could show for the first time the important role of IL-6 in predicting the outcome during intensive care treatment.

The retrospective study design might be a limitation. Even if our patients are treated by standard operating procedures, we cannot rule out that some results have been influenced by modified treatment due to peri-operative complications. Furthermore, the study refers to a single centre regional database, thus our results need to be further evaluated in other institutions and countries. It has been shown that both additive and logistic EuroScore were predictive for the outcome after cardiac surgery [37]. For our study, we used the additive EuroScore since it is very easy to use in clinical practice. Thus, we cannot rule out that the use of the logistic EuroScore may lead to different results. A significant correlation of NT-BNP and the EuroScore has been shown in the past [38]. To minimize any interferences in our statistical model, we decided not to include this parameter in our analyses. It might be possible that analysing cardiac-related biomarkers as NT-BNP or troponin might be also useful to predict the course in ICU. Finally, we focused on the evaluation of IL-6 as an inflammatory marker, therefore we cannot rule out that analysing other inflammatory markers (e.g., C-reactive protein, procalcitonin or white blood cell count) may lead to different results.

## Conclusion and outlook

Especially in the context of the COVID-19-pandemia but already before, allocation of ICU capacities and efficient resource allocation are important topics not just limited to cardiac surgery. Therefore, both pre-operative risk evaluation and postoperative outcome estimation are important aspects not only in the background of catastrophic situations, but also in regular major surgery settings. The results of our study support the eligibility of an existing Score (EuroScore) and the utilization of an easy to measure parameter (IL-6) for prediction of the course of cardiac surgery thus supporting the clinical aspects and resource discussions. In the context of newly developed scoring systems for this specific purpose, our data should be considered in the future advancements of these scoring systems to support clinical decision-making, allocation of resources and improvement of care for high-risk patients before and during intensive care treatment.

## Supplementary Information

**Additional file 1: Table S1.** Multilevel logistic regression models investigating possible predictors for duration of ventilation after cardiac surgery, including testing for interactions for EuroScore and IL-6 levels.

**Additional file 2: Table S2.** Multilevel logistic regression models investigating possible predictors for duration of stay in ICU after cardiac surgery, including testing for interactions for Euroscore and IL-6 levels. Baseline are patients being dismissed from ICU ≤ 2 days (compared to patients with being hospitalized on ICU > 2 days).

## Data Availability

The datasets used and/or analysed during the current study are available from the corresponding author on reasonable request (in compliance with data privacy).
