# Supplementary material for: EuroScore and IL-6 predict the course in ICU after cardiac surgery
Source: Eur J Med Res. 2021 Mar 26;26:29. doi: 10.1186/s40001-021-00501-1 (PMC7995398; doi:10.1186/s40001-021-00501-1)
Supplement: Supplementary file 1 — Additional file 1: Table S1. Multilevel logistic regression models investigating possible predictors for duration of ventilation after cardiac surgery, including testing for interactions for EuroScore and IL-6 levels. [file 40001_2021_501_MOESM1_ESM.docx]

|  |  | *multivariabel model*  *with interaction* | | |
| --- | --- | --- | --- | --- |
| *Variable* | *Category* | *OR* | *95% CI* | *p-value^j^* |
| **Age [years]^a^** | Linear | 1.01 | [1.00,1.02] | 0.01 |
| **Sex^b^** | Male | 1 | [1.00,1.00] |  |
|  | Female | 1.03 | [0.85,1.24] |  |
| **EF [%]^c^** | ≤ 45 | 1 | [1.00,1.00] |  |
|  | 46 - 64 | 0.71 | [0.56,0.91] |  |
|  | ≥ 65 | 0.69 | [0.56,0.85] |  |
| **EuroScore^d^** | 0-2 | 1 | [1.00,1.00] |  |
|  | 3-5 | 0.91 | [0.55,1.51] |  |
|  | > 6 | 0.93 | [0.55,1.58] |  |
| **Creatinine [mg/dl]^e^** | < 1.2 | 1 | [1.00,1.00] |  |
|  | 1.2 – 2.1 | 1.55 | [1.30,1.85] |  |
|  | > 2.1 | 1.65 | [1.03,2.64] |  |
| **IL-6 [pg/ml]^f^** | Q1 (< 102) | 1 | [1.00,1.00] |  |
|  | Q2 (102 - 165) | 0.81 | [0.47,1.39] |  |
|  | Q3 (166 - 256) | 0.84 | [0.49,1.44] |  |
|  | Q4 (257 - 421) | 0.99 | [0.59,1.65] |  |
|  | Q5 (> 421) | 0.73 | [0.43,1.24] |  |
| **CPB [min]^g^** | Q1 (< 72) | 1 | [1.00,1.00] |  |
|  | Q2 (72 - 85) | 1.15 | [0.90,1.48] |  |
|  | Q3 (86 - 100) | 0.89 | [0.69,1.14] |  |
|  | Q4 (101 - 124) | 1.03 | [0.80,1.32] |  |
|  | Q5 (> 124) | 1.87 | [1.44,2.44] |  |
| **Operation^h^** | CABG | 1 | [1.00,1.00] |  |
|  | AV | 0.88 | [0.72,1.08] |  |
|  | CABG + AV | 0.92 | [0.69,1.21] |  |
|  | MV | 1.25 | [0.93,1.68] |  |
|  | MV + TV | 2.21 | [1.10,4.43] |  |
| **Interaction**  **EuroScore^d /^ IL-6 [pg/ml]^f^** | 3-5/ Q1 (< 102) | 1 | [1.00,1.00] |  |
|  | 3-5/ Q2 (102 - 165) | 1.62 | [0.83,3.14] |  |
|  | 3-5/ Q3 (166 - 256) | 1.26 | [0.65,2.43] |  |
|  | 3-5/ Q4 (257 - 421) | 1.05 | [0.55,2.00] |  |
|  | 3-5/ Q5 (> 421) | 2.15 | [1.12,4.11] |  |
|  | > 6/ Q1 (< 102) | 1 | [1.00,1.00] |  |
|  | > 6/ Q2 (102 - 165) | 2.43 | [1.23,4.80] |  |
|  | > 6/ Q3 (166 - 256) | 2.39 | [1.21,4.74] |  |
|  | > 6/ Q4 (257 - 421) | 1.78 | [0.90,3.53] |  |
|  | > 6/ Q5 (> 421) | 3.9 | [1.93,7.86] |  |

**Table S2:** Multilevel logistic regression models investigating possible predictors for duration of stay in ICU after cardiac surgery, including testing for interactions for Euroscore and IL-6 levels. Baseline are patients being dismissed from ICU ≤ 2d (compared to patients with being hospitalized on ICU > 2d).

^a^Age is analyzed as linear variable.

^b^The reference category are males.

^c^Ejection fraction (EF) displayed in quintiles (resulting in 3 groups), reference category are values ≤ 45.

^d^EuroScore analyzed in three groups according to guidelines: mild/moderate/severe risk. The reference category is the lowest score.

^e^Creatinine displayed in three groups. The reference category are lowest values.

^f^IL-6 is displayed in Q (quintiles). The reference category is the first quintile.

^g^CPB is displayed in Q (quintiles). The reference category is the first quintile.

^h^Reference category is CABG.

^j^p-value: from likelihood ratio test comparing the regression models with (model above) and without interaction.

**OR:** odds ratio, **CI:** confidence interval, **EF:** ejection fraction, **CPB:** cardiopulmonary bypass, **CABG:** coronary artery bypass grafting, **AV:** aortic valve surgery, **CABG + AV:** combined operation of aortic valve and coronary artery bypass grafting, **MV:** mitral valve surgery, **MV + TV:** combined operation of mitral and tricuspid valve.
