# Supplementary material for: EuroScore and IL-6 predict the course in ICU after cardiac surgery
Source: Eur J Med Res. 2021 Mar 26;26:29. doi: 10.1186/s40001-021-00501-1 (PMC7995398; doi:10.1186/s40001-021-00501-1)
Supplement: Supplementary file 2 — Additional file 2: Table S2. Multilevel logistic regression models investigating possible predictors for duration of stay in ICU after cardiac surgery, including testing for interactions for Euroscore and IL-6 levels. Baseline are patients being dismissed from ICU ≤ 2 days (compared to patients with being hospitalized on ICU > 2 days). [file 40001_2021_501_MOESM2_ESM.docx]

|  |  | *multivariabel model*  *with interaction* | | |
| --- | --- | --- | --- | --- |
| *Variable* | *Category* | *OR* | *95% CI* | *p-value^j^* |
| **Age [years]^a^** | Linear | 1.01 | [1.00,1.02] | 0.003 |
| **Sex^b^** | Male | 1 | [1.00,1.00] |  |
|  | Female | 1.14 | [0.95,1.38] |  |
| **EF [%]^c^** | ≤ 45 | 1 | [1.00,1.00] |  |
|  | 46 - 64 | 1.04 | [0.82,1.33] |  |
|  | ≥ 65 | 0.78 | [0.63,0.96] |  |
| **EuroScore^d^** | 0-2 | 1 | [1.00,1.00] |  |
|  | 3-5 | 0.46 | [0.26,0.80] |  |
|  | > 6 | 0.89 | [0.52,1.53] |  |
| **Creatinine [mg/dl]^e^** | < 1.2 | 1 | [1.00,1.00] |  |
|  | 1.2 – 2.1 | 1.55 | [1.30,1.85] |  |
|  | > 2.1 | 1.91 | [1.18,3.09] |  |
| **IL-6 [pg/ml]^f^** | Q1 (< 102) | 1 | [1.00,1.00] |  |
|  | Q2 (102 - 165) | 0.46 | [0.26,0.80] |  |
|  | Q3 (166 - 256) | 0.89 | [0.52,1.53] |  |
|  | Q4 (257 - 421) | 0.87 | [0.52,1.45] |  |
|  | Q5 (> 421) | 1.03 | [0.61,1.74] |  |
| **CPB [min]^g^** | Q1 (< 72) | 1 | [1.00,1.00] |  |
|  | Q2 (72 - 85) | 0.97 | [0.76,1.24] |  |
|  | Q3 (86 - 100) | 0.97 | [0.75,1.24] |  |
|  | Q4 (101 - 124) | 1.08 | [0.84,1.39] |  |
|  | Q5 (> 124) | 1.62 | [1.24,2.11] |  |
| **Operation^h^** | CABG | 1 | [1.00,1.00] |  |
|  | AV | 1.02 | [0.84,1.24] |  |
|  | CABG + AV | 1.12 | [0.84,1.47] |  |
|  | MV | 1.08 | [0.80,1.46] |  |
|  | MV + TV | 1.84 | [0.93,3.64] |  |
| **Interaction**  **EuroScore^d /^ IL-6 [pg/ml]^f^** | 3-5/ Q1 (< 102) | 1 | [1.00,1.00] |  |
|  | 3-5/ Q2 (102 - 165) | 2.36 | [1.19,4.67] |  |
|  | 3-5/ Q3 (166 - 256) | 1.4 | [0.73,2.69] |  |
|  | 3-5/ Q4 (257 - 421) | 1.24 | [0.66,2.35] |  |
|  | 3-5/ Q5 (> 421) | 1.58 | [0.84,2.98] |  |
|  | > 6/ Q1 (< 102) | 1 | [1.00,1.00] |  |
|  | > 6/ Q2 (102 - 165) | 4.58 | [2.27,9.22] |  |
|  | > 6/ Q3 (166 - 256) | 1.83 | [0.93,3.60] |  |
|  | > 6/ Q4 (257 - 421) | 2.8 | [1.40,5.59] |  |
|  | > 6/ Q5 (> 421) | 2.52 | [1.26,5.03] |  |

**Table S1:** Multilevel logistic regression models investigating possible predictors for duration of ventilation after cardiac surgery, including testing for interactions for EuroScore and IL-6 levels.

^a^Age is analysed as linear variable.

^b^The reference category are males.

^c^Ejection fraction (EF) displayed in quintiles (resulting in 3 groups), reference category are values ≤ 45.

^d^EuroScore analysed in three groups according to guidelines: mild/moderate/severe risk. The reference category is the lowest score.

^e^Creatinine displayed in three groups. The reference category are lowest values.

^f^IL-6 is displayed in Q (quintiles). The reference category is the first quintile.

^g^CPB is displayed in Q (quintiles). The reference category is the first quintile.

^h^Reference category is CABG

^j^p-value: from likelihood ratio test comparing the regression models with (model above) and without interaction

**OR:** odds ratio, **CI:** confidence interval, **EF:** ejection fraction, **CPB:** cardiopulmonary bypass, **CABG:** coronary artery bypass grafting, **AV:** aortic valve surgery, **CABG + AV:** combined operation of aortic valve and coronary artery bypass grafting, **MV:** mitral valve surgery, **MV + TV:** combined operation of mitral and tricuspid valve.
